# Role of Geminin as a Tool for Augmenting Accurate Diagnosis of Cervical Neoplasia

**DOI:** 10.7759/cureus.56864

**Published:** 2024-03-25

**Authors:** Nilajkumar D Bagde, Madhuri N Bagde, Sarita Agrawal, Prasanta Nayak, Sanjay Singh Negi, Sarita Rajbhar, Nighat Hussain

**Affiliations:** 1 Obstetrics and Gynaecology, All India Institute of Medical Sciences Raipur, Raipur, IND; 2 Obstetrics and Gynaecology, Raipur Institute of Medical Sciences, Raipur, IND; 3 Obstetrics and Gynaecology, Oasis Fertility Hospital, Bhubaneswar, IND; 4 Microbiology, All India Institute of Medical Sciences Raipur, Raipur, IND; 5 Pathology and Laboratory Medicine, All India Institute of Medical Sciences Raipur, Raipur, IND

**Keywords:** human papillomavirus (hpv), immunohistochemistry staining, dna replication, diagnosis, tumor marker, cervical intraepithelial neoplasia, cervical cancer, hpv, pap smear, geminin

## Abstract

Aim: To determine the role of geminin as a tool for differentiating various types of cervical intraepithelial neoplasia (CIN) and cervical carcinoma (CC). Methods: Seventy women newly diagnosed with CIN or CC undergoing cervical biopsy were included; their clinical profile, human papilloma virus (HPV) positivity, and colposcopy findings were noted, and biopsy tissue was analyzed for geminin content. Results: On geminin immunohistochemistry, 100% of women with CIN3 and 96.29% of women with CC had geminin two plus or more. When analyzed as ordinal variables, there was a significant correlation (spearman’s rho 0.35, p 0.01) between geminin and biopsy results (CIN1, CIN2, CIN3, and CC). Conclusions: Screening tests for cervical cancer, like conventional pap smears, liquid-based pap smears, and triaging with HPV, have limitations. It is important to be able to differentiate between high-grade lesions, invasive cancer, and low-grade lesions. The detection of geminin in these cells may aid in the confirmation of the diagnosis and ensure adequate treatment. Cervical intraepithelial lesions and carcinoma cervix demonstrated a correlation between increased geminin expression in CIN1 vs. CC and CIN2 vs. CC. Geminin may be a potential surrogate marker for higher-grade cervical lesions, and further research is needed to corroborate evidence in this direction.

## Introduction

Precise duplication of chromosomal deoxyribonucleic acid (DNA) is a vital event in cell division. DNA replication occurs during the 'S' phase, which is different from the ‘M’ phase, during which chromosomes segregate and cells divide. It is vital for the genome to undergo complete replication only once during a single cycle, and no region should be replicated more than once. This is ensured by the replication licensing system [1]. A DNA helicase-minichromosome maintenance 2-7 (MCM 2-7) complex needs to be loaded on the replicating system to initiate replication. This process is called licensing of DNA and occurs several hours before origins are activated to fire for replication [2].

MCM recruitment is followed by origin recognition complex (ORC)-mediated binding of Cdc6 and Cdt1, which form pre-recognition complexes (PreRC) and initiate DNA licensing. Uncontrolled Cdt1 accumulation leads to multiple DNA replication events.

Geminin is an unstable tetramer protein of 209 amino acids that is existent during the S-G2-M phases and destroyed at the transition of metaphase to anaphase [3,4]. It binds Cdt1, averting the loading of MCM complexes to the origin and inhibiting licensing [4,5]. Thus, it regulates Cdt1 levels and safeguards eukaryotic cell replication [6]. Studies also show that geminin levels are crucial for both negative and positive regulation of PreRC complex formation. It allows Cdt1 accumulation in the G2-M phase and positively regulates PreRC while simultaneously preventing origins from getting relicensed in the S-G2 phase [3,5]. Geminin also promotes the termination of DNA replication forks, preventing re-replication [7]. However, despite impeding DNA re-replication, geminin is overexpressed in some tumor cells, and the high proliferation rates of certain tumors are associated with increased geminin levels [8].

In this study, we attempt to determine levels of geminin in women with cervical intraepithelial neoplasia and cervical carcinoma and determine if these levels are associated with increasing grades of disease. The objective was to determine the efficacy of geminin to differentiate between various types of cervical intraepithelial neoplasia and cervical cancer and its correlation with HPV subtypes.

## Materials and methods

Patient sampling and criteria

This was a cross-sectional study conducted in the Department of Obstetrics and Gynecology, All India Institute of Medical Sciences, Raipur. Due permission was obtained from the institute ethical committee (Number: AIIMSRPR/IEC/2019/317) for the project. It was an intramural project funded by the institution itself. All women who were diagnosed with CIN or cervical carcinoma in the gynecology cancer clinic and consented to study between 2021 and 2022 were included.

Inclusion criteria

All freshly diagnosed cases of CIN, or cancer cervix, diagnosed with the disease for the first time in the institute during the duration of the study were included.

Exclusion criteria

All those cases with CIN, or cancer cervix, who had undergone treatment were excluded. Seventy women attending the gynecology cancer clinic, freshly diagnosed as CIN or cancer cervix during the vaginal examination or colposcopy, and who underwent cervical biopsy were included after informed consent. All women were included on a first-come, first-served basis. Colposcopy is routinely performed in the OPD of the OB-GYN department and was performed wherever it was indicated. A detailed history was elicited about complaints like persistent vaginal discharge, metrorrhagia, postcoital bleeding, intermenstrual bleeding, postmenopausal bleeding, or any other. If possible, Reid’s score was noted during colposcopy and categorized as 0-2: CIN1, 3-5: CIN1/2, 6-8: CIN2/3. HPV reports were noted if available. A cervical biopsy was performed using punch biopsy forceps and evaluated for histopathology and geminin testing. The tissue diagnosis on biopsy was reviewed by two independent pathologists. The colposcopy findings, biopsy report, and other related information were recorded in a predesigned proforma. Biopsy reports were reported as CIN1: one-third of the epithelium involved; CIN2: two-thirds involved; CIN3: full thickness of the epithelium involved but basement membrane intact; invasive cancer: basement membrane invaded by malignant cells.

Geminin detection

**Figure 1:**
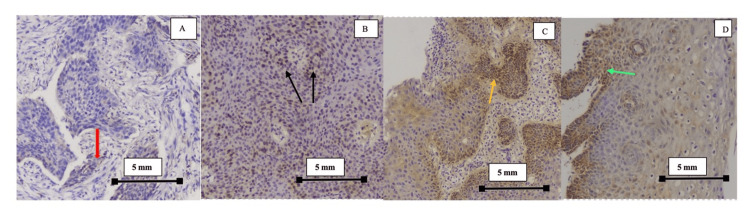
Immunohistochemistry: Geminin staining and scoring Immunohistochemistry of cervical biopsy samples with counterstain hematoxylin. All images are at 10x magnification, as mentioned in the scale of each image. Image A: squamous cell carcinoma with geminin positivity in less than 5% of cells, consistent with a score of 0 (red arrow). Image B: atypical squamous cells in full thickness of the epithelium in CIN show geminin positivity in 5-25% of cells, consistent with score 1 (black arrows). Image C: atypical squamous cell in the lower one-third of the epithelium in CIN, show geminin positivity in 25-50% of cells, consistent with score 2 (yellow arrow). Image D: atypical squamous cells in the lower two-thirds of the epithelium in CIN, show geminin positivity in >50% of cells (green arrow), consistent with score 3.

Cervical biopsy tissue was used for the detection of geminin expression. The technique used included immunohistochemistry analysis of all formalin-fixed paraffin-embedded sections cut at 5mm thickness and then dewaxed through xylene and dehydrated with graded ethanol. Sections were treated with a 3% hydrogen peroxide solution for 20 minutes to block endogenous peroxidase activity. Antigen retrieval was performed in 10mMol/L of citrate buffer. They were incubated over night with a monoclonal mouse antibody to geminin. Subsequently, tissue sections were counterstained with hematoxylin and examined by light microscopy. For negative control, substitution of primary antibodies with tissue biopsy samples was run simultaneously. Geminin levels were reported (Figure 1) as 0: where < 5% cells were negative; 1 plus: where 6-25% cells stained positive; 2 plus: where 26-50% of cells stained positive; and 3 plus: where more than 50% of cells stained positive. HPV DNA detection was done by the hybrid capture II technique.

Statistical analysis

Analysis was done using Stata version 14. Means and standard deviations were reported for continuous variables, and descriptive statistics for baseline data. Geminin, body mass index, and cervical biopsy were analyzed as ordinal variables. Individual biopsy reports were additionally analyzed as categorical. HPV was analyzed as categorical. Ordinal data was analyzed by Spearman rank correlation, and Kruskal-Wallis’s equality of population rank was used for ordinal data. HPV status, biopsy reports, and levels of geminin expression were correlated. All tests were two-tailed with a significance level of p < 0.05.

## Results

A total of 70 women participated in the study and were evaluated for various parameters. 

Demographic parameters

The mean age of participants was 47.51 +/- 11.90 years and ranged from 27 to 76 years. The average duration of marriage was 27.5 years, ranging from 1 to 60 years. 54.25% of women were either illiterate or educated until primary school (Table 1).

**Table 1:** Population characteristics

Parameter	Percentage
Education	
Illiterate	32.85
Primary school	21.40
Middle school	05.71
Secondary school	24.28
Higher secondary school	10.00
Graduate	05.71
Age at marriage	
16-20 years	11.42
21-25 years	58.57
26-30 years	24.28
>30 years	05.71
Age at first childbirth (data available for N = 51 women)	
< 20 years	29.40
20-24 years	60.78
25-29 years	09.80
Interpregnancy interval (data not available for 6 women and 2 were primipara, N= 62)	
< 2 years	38.57
2-3 years	37.14
3-4 years	11.42
>= 4 years	01.42
Duration since menopause	
Not attained menopause	54.28
1-5 years	07.14
6-10 years	20.00
11-15 years	11.42
16-20 years	05.71
>21 years	01.42

Clinical features, HPV, and Reid’s score

White discharge was reported by 42.85% of women; 7.14% had menorrhagia; 4.28% had intermenstrual bleeds; 10% had postcoital bleeds; and 21.42% had postmenopausal bleeding. 

It was found that the expression of geminin in cervical biopsy tissue was not related to age (Kendall’s tb = 0.13, p 0.22). Geminin levels were also unrelated to age at marriage, age at first childbirth, duration of married life, parity, and BMI (body mass index, categorized as per the Indian guidelines [9]) (p > 0.05 in all).

HPV reports were available for 35 women. These included positivity for HPV 16, 18, and 33, either as a single or multiple virus positivity. The HPV status did not correlate with geminin levels (Kruskal-Wallis c2 = 0.23, p 0.62) for either HPV positive status or individual virus positivity (p = 0.73 for HPV 16, 0.65 for HPV 18, and 0.14 for HPV 33).

Reid’s score was available for 35 women that underwent colposcopy. The mean score was 5.14 + 2.19. According to colposcopy Reid’s score, 14.29% had CIN1, 38.10 had CIN1/2, and 47.62% had CIN2/3. There was no correlation between Reid’s score and geminin levels (Spearman’s rho 0.38, p 0.08) in the biopsy specimens.

Cervical biopsy diagnosis and geminin

Cervical carcinoma (CC) was observed on cervical biopsy in 51.42%, and 48.56% had CIN (CIN1 in 27.14%, CIN2 in 15.71%, and CIN3 in 5.71%). On geminin immunohistochemistry, 100% of women with CIN3 and 96.29% of women with CC had geminin staining of two plus or more (Table 2). 

**Table 2:** Geminin levels in cervical biopsy reports. *CIN: Cervical intraepithelial neoplasia, #CC: Cervical carcinoma

Cervical biopsy report	Geminin (percentage)			
	0	1 plus	2 plus	3 plus
CIN*1	35.71	14.29	14.29	35.71
CIN2	25	12.5	50	12.5
CIN3	0	0	100	0
CC^#^	0.00	3.70	51.85	44.44

On comparing geminin level vs. CIN1, CIN2, CIN3, and CC as categorical variables, no correlation was observed between geminin levels and CIN1, CIN2, and CIN3 individually compared to the other three. However, there was a significant difference between geminin and CC (Kruskal-Wallis’s equality of population rank c2 6.38, p 0.01) compared to CIN1, CIN2, and CIN3 combined (Table 3). 

**Table 3:** Comparison of geminin levels and cervical biopsy results: Combined lesions *CIN: Cervical intraepithelial neoplasia, #CC: Cervical carcinoma

Cervical biopsy results and geminin	Kruskal-Wallis’s equality of population rank c^2^	p-value
Geminin in CIN1 vs. CIN2, CIN3, CC (combined)	1.99	0.15
Geminin in CIN2 vs. CIN1, CIN3, CC (combined)	2.23	0.13
Geminin in CIN3 vs. CIN1, CIN2, CC (combined)	0.17	0.68
Geminin in CC vs. CIN1, CIN2, CIN3 (combined)	6.38	0.01

When analyzed as ordinal variables, there was a significant correlation (Spearman’s rho 0.35, p 0.01) between geminin and histopathological biopsy results (CIN1, CIN2, CIN3, and CC) (Table 4). 

**Table 4:** Comparison of geminin levels and cervical biopsy results: Individual lesions *CIN: Cervical intraepithelial neoplasia; #CC: Cervical carcinoma

Cervical biopsy results and geminin	Spearman’s rho	p-value
CIN*1 vs. CIN2	-0.02	0.91
CIN1 vs. CIN3	0.09	0.70
CIN1 vs. CC^#^	0.31	0.04
CIN2 vs. CIN3	0.22	0.50
CIN2 vs. CC	0.40	0.01
CIN3 vs. CC	0.24	0.19

## Discussion

Cervical carcinoma is a leading cause of cancer-related mortality. Screening tests like conventional pap smears, liquid-based pap smears, and triaging with HPV have limitations. Triage testing, combining HPV with cytology and p16/K67 dual stain, is introduced to accurately assign risk and referral for further testing after risk quantification [10]. It is important to be able to differentiate between high-grade lesions, invasive cancer, and low-grade lesions. The detection of geminin in these cells may aid in strengthening the accuracy of the diagnosis.

Geminin is associated with cellular senescence, and Cdt1 and geminin are shown to be down-regulated in human fibroblasts under stress-induced senescent changes [11]. However, there is no literature that studies geminin levels with age. The behavior of geminin levels with increasing individual age is not yet studied in cells, more so in cervical tissue. In this study, we did not find any association between geminin in cervical tissue and increasing age. Similarly, we failed to find an association between geminin and age at marriage, age at first childbirth, duration of married life, parity, and BMI. All of the above factors have been linked to CIN, or cervical carcinoma [12-14]. In this study, geminin is indifferent to these factors and appears to vary only with CIN status. No studies were found that examined the relationship between these factors and geminin levels in CIN or carcinoma cervix.

Reid’s score is used to assess grades of CIN in colposcopy [15]. Considering that geminin varies with increasing levels of CIN and also carcinoma, it was expected that it would show a similar result for Reid’s score, too. However, geminin levels did not correlate with Reid’s score in this study. The reason for this may have been the poor correlation of Reid’s score with cervical biopsy results in this study. Reid’s score was not available for frank carcinoma as a direct biopsy was done where geminin levels were high. We did not find any studies that compare Reid’s score and geminin levels in cervical biopsy specimens. 

In this study, geminin levels were correlated with cervical intraepithelial lesions and cervical carcinoma (Table 3) on histopathology. There was a positive correlation between geminin levels in women with CC compared to those with CIN1 and also in CC compared to CIN2. Levels were not significantly different in CIN3 compared to CC. However, more women with CIN3 or CC had geminin positivity staining as compared to those with CIN1 or CIN2 (Table 2), and the correlation was significantly higher for CC compared to CIN1 or CIN2 (Table 3). Zheng et al. [16] also found HPV 16/18 positivity status, expression of geminin, presence of squamocolumnar junction, and size of lesion as independent predictors for differentiating between low-grade squamous intraepithelial lesions and high-grade squamous intraepithelial lesions. They reported HPV 16/18 status and geminin as the most significant associations. Hence, the presence of elevated geminin may indicate a higher grade of lesion (CIN3 or CC) and warrant more detailed evaluation and management. Xing et al. [17] reported a significantly higher expression of geminin in CIN2-3 compared to CIN1 and propose that it may be used as a surrogate marker for higher lesions. They reported a sensitivity of 95%, a positive predictive value of 85.7%, and an accuracy of 88.2%. Geminin levels were found to increase with the grade of the lesion and also change during the progression of LSIL/CIN1 to HSIL/CIN2-3 [16].

Geminin has been proposed to regulate gene expression and replication of DNA across evolution [18]. It has been shown to selectively couple transcription factor forkhead box O3 to histone deacetylase and block its activity, ultimately downregulating its target Dicer, an RNase that suppresses metastasis in breast cancer. Also, increased expression of geminin in proliferating lymphocytes and epithelial cells indicates its positive correlation with cell proliferation, though it itself inhibits the same [19]. Elevated geminin levels may also serve as a marker for metastatic disease [20], and this needs to be evaluated in cervical carcinoma. 

HPV positivity did not correlate with geminin in this study, which was a limitation. It may be attributable to the small sample size. Reid Index was also not available for all women, as women with carcinoma underwent direct biopsy. 

## Conclusions

Geminin is a tetramer DNA replication inhibitor that exists during the S-G2-M phases of the cell cycle. Its overexpression has been reported in some tumor cells. Cervical intraepithelial lesions and carcinoma cervix demonstrated a correlation between increased geminin expression in CC vs. CIN1 and CC vs. CIN2 in this study. Also, geminin expression was higher in cervical cancer than in any of the three premalignant lesions (CIN1, CIN2, and CIN3). Thus, geminin may prove to be a marker for higher-grade cervical lesions. Hence, its role as a potential surrogate marker for cervical carcinoma needs to be further researched to corroborate evidence in this direction. 
